# Biosolid as an alternative source of nutrients in chrysanthemum cultivation

**DOI:** 10.1038/s41598-024-66040-x

**Published:** 2024-09-04

**Authors:** Frederico Luiz Pereira, Ursuléia Aparecida de Oliveira, Márcio Donizetti de Andrade, Felipe Campos Figueiredo, Breno Régis Santos, Marília Carvalho, Sandro Barbosa

**Affiliations:** 1 Instituto de Ciências da Natureza, Universidade Federal de Alfenas, Alfenas, MG 37130-000 Brazil; 2 Laboratório de Solos e Tecido Vegetal, Cooperativa Regional de Cafeicultores em Guaxupé, Guaxupé, MG 37800-000 Brazil; 3 Laboratório de Biotecnologia e Cultura de Tecidos, Instituto Federal de Educação, Ciência e Tecnologia do Sul de Minas Gerais – Campus Muzambinho, Muzambinho, MG 37890-000 Brazil

**Keywords:** Environmental sciences, Natural hazards

## Abstract

The objective was to evaluate the biosolids as an alternative source of nutrients in the production of chrysanthemums by adding increasing doses to the cultivation substrate. The experimental design was in blocks with 6 treatments and 5 replications. The treatments consisted of the mixture (commercial substrate + biosolid) at the concentrations: 20%, 40%, 60% and 80% of biosolid + two controls (100% of biosolid and 100% of substrate). The experiment was conducted in a greenhouse for 90 days. Physiological parameters, number of flower buds, dry biomass and nutrient accumulation were evaluated. Physiological parameters were evaluated using the Infrared Gas Analyzer. The number of flower buds was evaluated by counting. Biomass was determined after drying the structures and then calculated the accumulation of nutrients. A total of 90 plants were evaluated. Concentrations of up to 40% of biosolid promoted a greater number of flower buds, dry biomass and nutrient accumulation. Concentrations above 60% lower number of buds, biomass increment and nutrient accumulation. It is concluded that the biosolid has potential as an alternative source of nutrients in the cultivation of chrysanthemums, indicating concentrations of up to 40% and the nutrient content of each batch generated must be verified.

## Introduction

Few studies deal with the nutrition of ornamental plants, especially in the cultivation of chrysanthemums. As they are plants with possibilities of cultivation in a greenhouse, substrates are used, and they have to offer good physical and nutritional conditions.

The biosolid, waste generated in a sewage treatment plant through the treatment of sewage sludge, has a nutrient rich composition, containing phosphorus (P), calcium (Ca), magnesium (Mg), surfur (S), boron (B), iron (Fe), zinc (Zn), copper (Cu) among others, essential to plants and their use can reduce or eliminate the need for inorganic fertilizers^1,2^ and appers as an alternative source of nutrient supply, since its inadequate disposal in the environment can cause serious problems. The growth of the world population, estimated at 9.8 billion people by 2050^3,4^ should cause an increase in the generation of sewage sludge, with an approximate production of 13 million tons, requiring a suitable destination^5^. The adequate use of biosolids in ornamental crops may increase the demand for biosolids in greenhouse crops and ornamental nurseries, also changing the negative perception of waste. Biosolids have been the subject of studies, due to their ability to provide nutrients and boost plant growth. Organic fertilizers, compared to inorganic ones, are important in promoting the gradual release of nutrients to plants as they decompose and stimulate plant development^6^. Increases in substrate fertility with the addition of biosolids were observed, with emphasis on phosphorus, nitrogen and calcium contents^7^, especially P, where biosolids have the potential to supply this nutrient, in addition to improving its bioavailability in soils fertilized with this residue or derived products^8^. Studies with ornamental plants show growth of 10 to 60% of the plants with the use of biosolids^9^. Studies indicate that at concentrations of 40 to 60% of biosolids in the substrate when cultivating Syagrus romanzoffiana, the species reached a height of 30 to 40 cm^10^. Knowledge of the nutritional needs of the crop is intrinsically related to fertilization, which favors quality, yield and longevity of the inflorescences and the plant^11^.

The commercialization of chrysanthemums is directly related to the size and quality of leaves, stems and flowers, and the success for the production of plants with these characteristics is also associated with nutritional conditions^12^. A sustainable flower production requires an ideal fertilizer, to achieve a yield with high ornamental value and to reduce production costs^13^. In the ornamental production sector, chrysanthemum is of great interest in the flower market and is highly demanded by the consumer due to the beauty and durability of its inflorescences. It is considered a relatively easy growing plant, but it requires a great cultivation technology, besides a substrate that offers good physical and chemical characteristics^14^. Considering the last 3 decades, it was the most cultivated ornamental species, the 3rd in potted cultivation and with a 7% share of the potted flower market. In addition, the chrysanthemum is produced during all months of the year, the plants respond precisely to photoperiod control (floral induction) being classified as a short-day plant^15^. There are limited data on the use of biosolids in ornamental plants and, in some countries, biosolids are used in the horticultural sector, where a study carried out indicates the effects of different proportions of biosolids on *Ixora chinensis*, *Schefflera heptaphylla*, and *Hibiscus rosa-sinensis*^5^. The difficulty in the cultivation of chrysanthemums is in quantifying the doses of fertilizers to be applied, according to the nutritional needs. In general, producers use unbalanced doses, which interfere with the plant production potential and longevity^16^.

The dynamics of nutrient accumulation in dry biomass throughout the growing season characterizes the nutrient uptake and quantifies the nutritional requirements and the appropriate times for fertilization. Thus, problems related to salinity or nutritional deficiency are avoided, as well as the possibility of correcting any deficiencies and evaluating nutritional status^17,18^. The accumulation of nutrients in chrysanthemums accompanies the production of dry biomass and is intensified during the development of flower buds and inflorescence opening^19^.

In studies involving the accumulation of nutrients in the ‘White Diamond’ chrysanthemum shoot, the authors observed the accumulation and concentration of nutrients in the order K > N > Ca > P > Mg > S (1425, 892, 184, 150, 110 and 59 mg plant^−1^) and Fe > Zn > B > Mn > Cu (2.3, 2.2, 1.7, 1.3, 0.21 mg plant^−1^)^11^. The sequence of accumulated macronutrients, in descending order, for cultivar ‘White Diamond’, is K > N > P^20^. Balanced doses of N, P and K increase plant growth, favoring the synthesis of peptide binding, protein and carbohydrate metabolism that are essential for the development of chrysanthemums^21^.

The uptake of N in chrysanthemums occurs gradually until flowering and tends to decrease after this phase^22^. The need for phosphorus in chrysanthemum plants is low and significantly lower than that of nitrogen^23^. Responses to changes caused by biosolids can improve the understanding of P uptake by crops and optimize the use of P-rich biosolids for sustainable agriculture^24^. As for potassium requirements, the requirements in the plant are high and this element favorably affects flower growth and color^25^. Considering the lack of information on the use of biosolids as a substrate and in nutrient supply, the objective of this study was to evaluate the production and nutrition of substrate grown ‘White Diamond’ chrysanthemums with biosolids.

## Results

### Macronutrient accumulation in chrysanthemum plants

**Figure 1:**
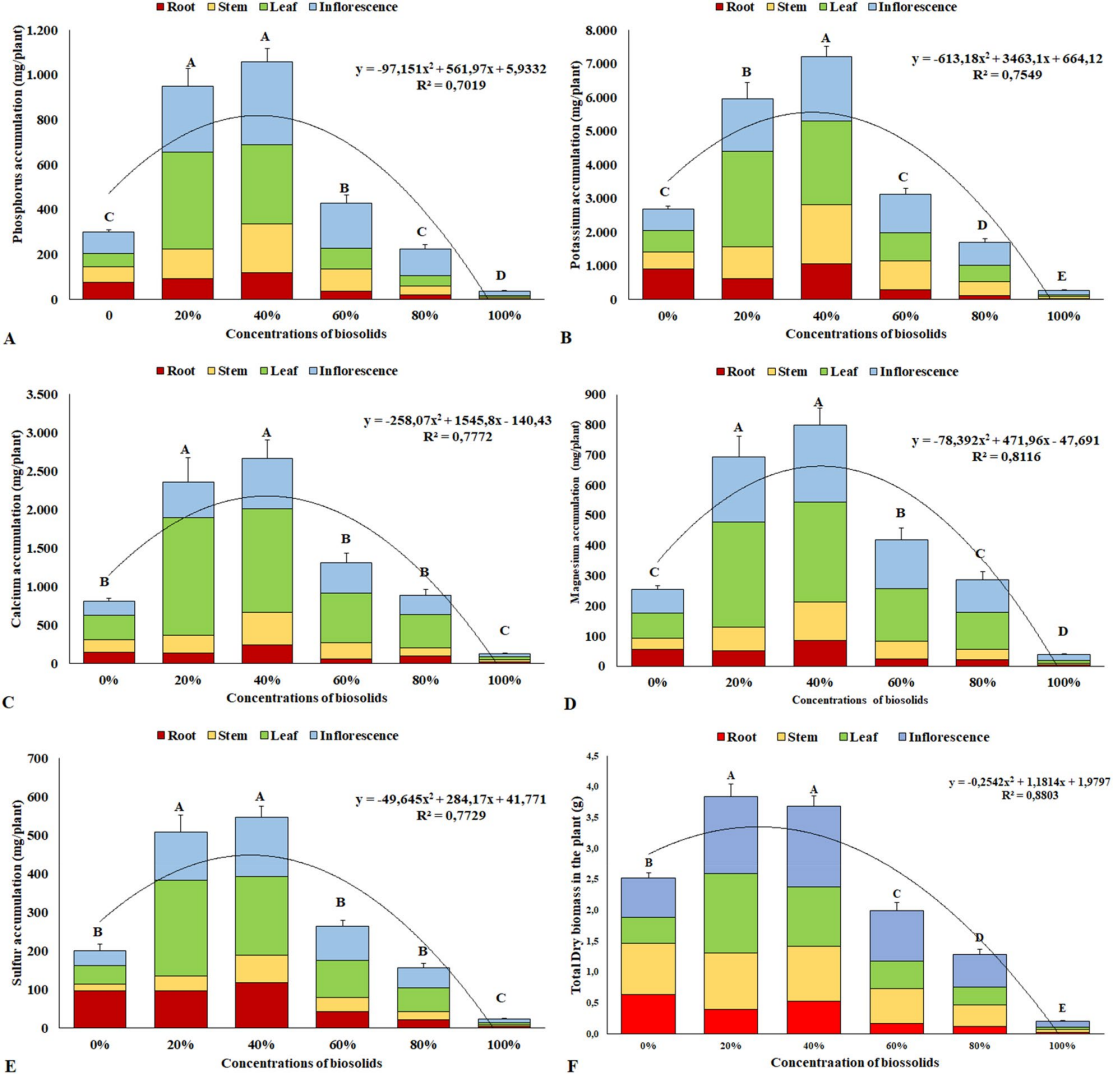
Total accumulation of macronutrients and dry biomass in chrysanthemum plants at 90 days of cultivation. Phosphorus (**A**); Potassium (**B**); Calcium (**C**); Magnesium (**D**); Sulfur (**E**); Total dry biomass (**F**). Same letters do not differ by the Scott–Knott test at 5% significance.

The results for the increment of total dry biomass in the chrysanthemum plants after 90 days of cultivation are presented in Fig. 1F. The addition of 20% and 40% biosolid yielded the largest macronutrient accumulations. The accumulation of macronutrients at 90 days of cultivation has the following order: K > Ca > P > Mg and S. Concentrations equal to or greater than 60% of biosolid reduce the total dry biomass.

**Figure 2:**
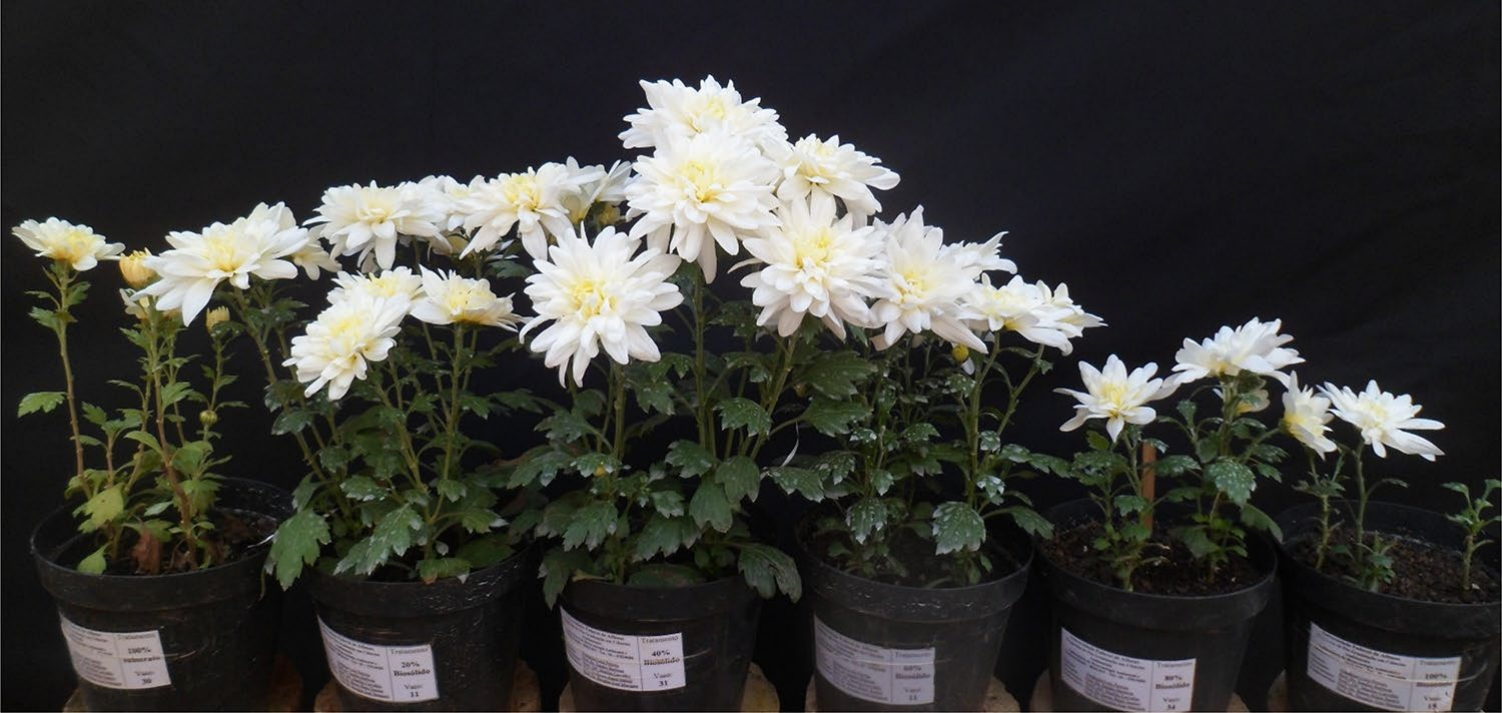
Chrysanthemum plants submitted to concentrations of 0, 20%, 40%, 60%, 80% and 100% of biosolid, respectively, at 90 days of cultivation.

Chrysanthemum plants grown with 20% and 40% biosolid accumulated more phosphorus than other biosolid concentrations (Fig. 1A). The accumulation in the leaves and inflorescences of plants at 90 days of cultivation stood out. For the concentration of 40% biosolids, accumulations of 1057 mg plant^−1^ of P were observed. In plants grown at concentrations of 80% and 100% biosolid, a lower P accumulation was observed, related to the smaller increase in dry biomass, due to the small root volume of these plants, evidencing plants with low growth and development in relation to the others, from the beginning of their cultivation (Fig. 2).

As for potassium, the concentration of 40% biosolid yielded the highest accumulation of this element in plants, 7218 mg plant^−1^, followed by the concentration of 20% biosolid, which led to an accumulation of 5962 mg plant^−1^, to the detriment of the others (Fig. 1B). At concentrations above 80% biosolid, the plants had lower potassium accumulation, possibly due to their lower root growth, as observed for P accumulation, and it could even be associated to a saline effect of these substrates, negatively affecting root growth. Figure 2F shows the increment in total dry biomass in chrysanthemum plants caused by the concentrations of up to 20% and 40% biosolid, to the detriment of the other concentrations and the substrate with the absence of biosolid, evidencing the results obtained by the accumulation of nutrients, especially phosphorus and potassium.

**Figure 3:**
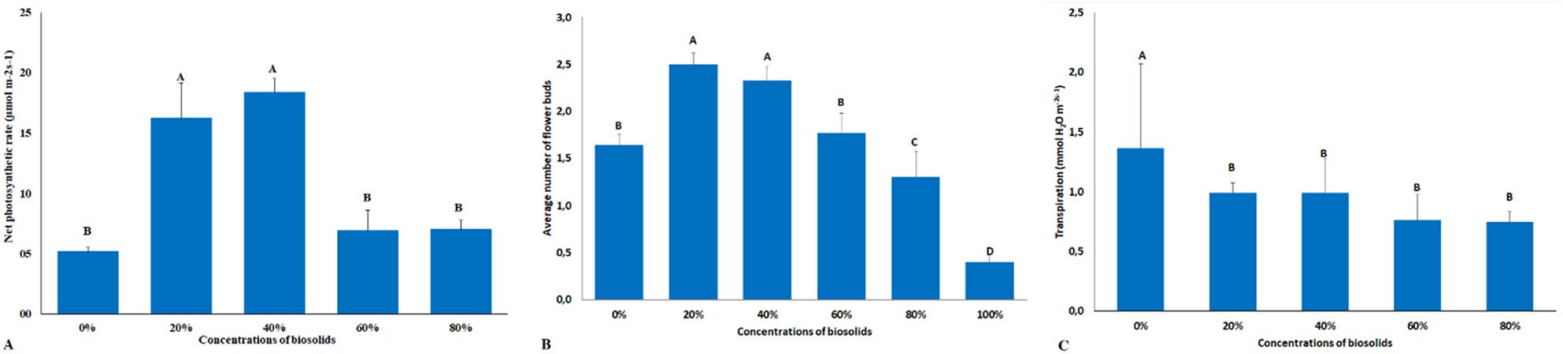
Net photosynthetic rate (**A**); Average number of flower buds (**B**); Transpiration (**C**) in chrysanthemum plants submitted to concentrations of 0, 20%, 40%, 60%, 80% and 100% of biosolid.

For calcium, concentrations of 20% and 40% biosolid led to higher accumulations, to the detriment of the others, especially in the leaves (Fig. 1C) since, at these concentrations, the available calcium amounts were higher, as they increased proportionally with the increase in biosolid concentration, which influenced the higher uptake by plants, once calcium is absorbed by mass flow through transpiration (Fig. 3C), thus accumulating more in the tissues.

Similarly, to the results observed for Ca accumulation, concentrations of 20% and 40% biosolid yielded greater accumulations of magnesium in the plants, with emphasis on the accumulation in the leaves (Fig. 1D), thus leading to a better production of photosynthetic pigments. The concentrations of 20% and 40% biosolids had the highest photosynthetic rates at 90 days of cultivation, in relation to the other concentrations and in the absence of biosolids (Fig. 3A). These results evidenced that the better availability and greater accumulation of elements in these plants guaranteed a better nutritional balance, which positively affected photosynthetic rates, noticed by the number of flower buds (Fig. 3B). Plants submitted to 20% and 40% biosolids showed a higher flower bud development indicating that, at these biosolid concentrations, the best supply of mineral elements was also more effective in yielding greater bud production.

For sulfur, there was a greater accumulation of the element at concentrations of 20% and 40% biosolid, to the detriment of the others (Fig. 1E). These results show the effective participation of biosolids in the sulfur supply to the plants.

### Micronutrient accumulation in chrysanthemum plants

Likewise, as observed for macronutrient accumulation, the concentrations of 20% and 40% biosolids yielded the greatest accumulations of micronutrients in the plants at 90 days of cultivation. The accumulation of micronutrients at the concentrations of 20% and 40% biosolid has the following order, respectively: Fe > Mo > B > Mn = Zn > Cu/Fe > Mo > B > Zn > Mn > Cu.

**Figure 4:**
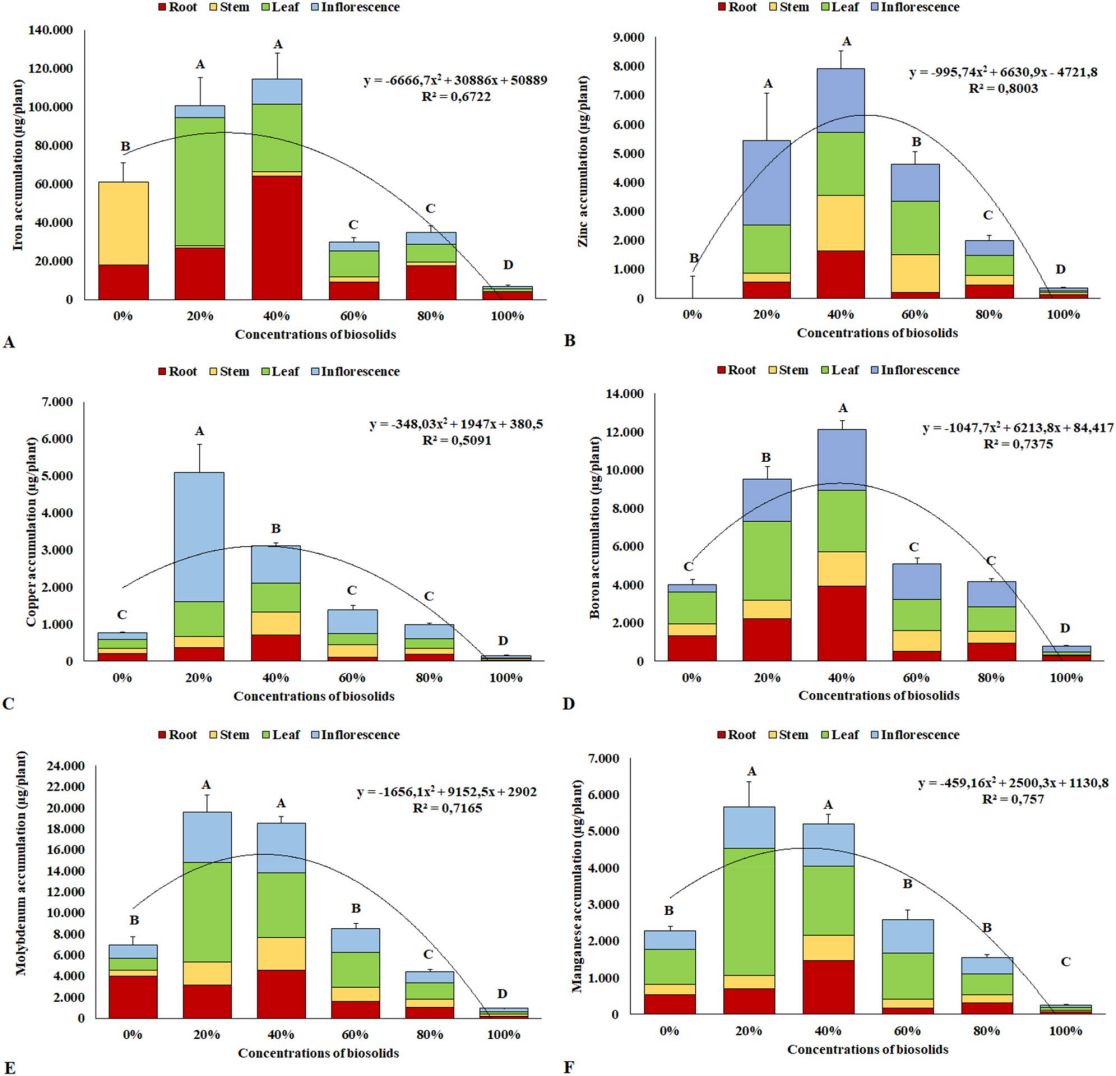
Total accumulation of micronutrients in the dry biomass of chrysanthemum plants at 90 days of cultivation. Iron (**A**); Zinc (**B**); Copper (**C**); Boron (**D**); Molybdenum (**E**); Manganese (**F**). Same letters do not differ by the Scott–Knott test at 5% significance.

In the case of iron, concentrations of 20% and 40% biosolids yielded a greater accumulation of the element, with emphasis on the accumulation in the roots and leaves (Fig. 4A). However, in spite of the levels higher than 80,000 μg plant^−1^ identified at concentrations of 20% to 40%, no symptoms were observed in these plants that could characterize Fe toxicity in the tissues, which allows to infer that chrysanthemum plants are tolerant to high salt accumulation.

For zinc, accumulations of the element in plants submitted to substrates with the addition of biosolids, especially the concentration of 40%, followed by the concentrations of 20% and 60% to the detriment of the others, were identified. In plants grown on substrates with the absence of biosolids, no element accumulation was identified, indicating that only the commercial substrate did not supply zinc to plants (Fig. 4B).

As for copper, the concentration of 20% biosolids showed plants with a higher accumulation of the element, mainly in the inflorescences of these plants, with concentrations of 5097 μg plant^−1^ (Fig. 4C).

For boron, a higher accumulation was observed at the concentration of 40% biosolid, followed by the concentration of 20% biosolid, emphasizing the accumulation in the roots and leaves (Fig. 4D). Despite its immobility in plant tissues, the accumulation of this element was observed in the roots, possibly related to the high concentrations of aluminum identified in plant roots.

As for molybdenum, there was a greater accumulation at the concentrations of 20% and 40% biosolids, to the detriment of the other concentrations and without the addition of biosolids (Fig. 4E), highlighting the accumulated in plant roots, leaves and inflorescences.

Similarly, to the other micronutrients, higher concentrations of manganese were observed at 20% and 40% biosolids, highlighting the accumulation in the leaves. Except for the concentration of 100% biosolid, no differences were observed in the accumulation of the element at other concentrations (Fig. 4F).

## Discussion

The accumulation of phosphorus found at the concentrations of 20% and 40% are much higher than the 40.9 mg plant^−1^ and 50.23 mg plant^−1^ found in two studies^16,20^, where the authors worked with fertigation, differently from this study, where no nutrients were added other than those present in the substrates. Concentrations of 5% sewage sludge and 95% soil increased P accumulation by 78% in chrysanthemum seedlings in relation to the control treatment, and levels of 5 mg plant^−1^ in another study^26^.

The potassium accumulation found in this study is lower than those found in a study using the same cultivar, where the authors observed the accumulation of 93,400 mg plant^−1^ potassium^20^. A sudden increase in potassium accumulation in flower buds, from 14.31 to 69.18 mg plant^−1^, was observed at 8 weeks of cultivation of fertigated chrysanthemums in another study^27^. However, the results are lower compared to this study. There are reports that chrysanthemum plants are highly demanding in potassium^28,29^, which is the most required nutrient when the flowering period approaches, from floral induction to its opening^30^. A potassium accumulation of 52,000 mg plant^−1^ is found during the three-month cycle of potted chrysanthemum^31^.

In evaluations of macronutrient contents in two chrysanthemum cultivars (‘Amarelo São Paulo’ and ‘Puritana’) through the supply of urban waste compost (CLU), together with carbonized rice husk (CAC), at concentrations of 100% CAC; 67% CAC + 33% CLU; 33% CAC + 67% CLU and 100% CLU, it was observed that potassium remained constant, independent of the concentration of CLU and CAC. The varieties did not show different concentrations for potassium, when submitted to different concentrations of CLU and CAC^29^. Concentrations above 80% biosolids affected root growth, as potassium can affect root growth due to the saline effect on the roots.

In relation to the accumulation of calcium found in this study, similar results were observed evaluating the effect of urban waste compost (CLU), together with carbonized rice husk (CAC). It was also observed that, at a concentration of 30% of CLU and 70% of CAC, there was a calcium increase in the leaves, above the minimum limits in two chrysanthemum cultivars^29^.

Considering the participation of calcium in the structure of the plant, its presence in the stem, especially in chrysanthemums, may be related to stem support, which is important for the closure and compaction of plant inflorescences in commercialization. Other effects related to the presence of calcium are reported^32^ where the authors studied the effect of calcium on the tolerance of *Lisianthus* by the application of alkaline irrigation water. The authors could verify that calcium contributes to the increase in plant tolerance to the alkalinity of soils/substrates, and that they showed improvements in growth and accumulation of dry biomass, which is an observation of this study.

In addition to the aforementioned functional activities, calcium has been reported as an element that improves abiotic stress tolerance in plants, including drought^33^ and even excessive boron concentrations^34^ as the plants accumulated high amounts of boron, mainly in the roots (Fig. 4D).

The major magnesium accumulation in the leaves is fundamental for the formation of photosynthetic pigments, and magnesium, together with nitrogen, is important in the structuring of chlorophyll molecules that act in the capture of photosynthesizing radiation^35^. Plants submitted to substrates with high calcium accumulation commonly have high amounts of magnesium^36^.

For iron accumulation, studies reported that Fe concentrations ranging from 10,000 to 1,500,000 μg kg^−1^ dry biomass can be found in plants; however, at concentrations above 80,000 μg kg^−1^, toxicity symptoms may already be observed^37,38^. Nevertheless, in spite of the levels higher than 80,000 μg plant^−1^ identified at concentrations of 20% to 40%, no symptoms were observed in plants that could characterize Fe toxicity in the tissues, which allows to infer that chrysanthemum plants are highly tolerant to salt accumulation.

In relation to the accumulations of zinc, high zinc concentrations present in biosolids contribute to a greater accumulation of this nutrient in plants, which was evident through this study^39^. Plants with phytoextraction capacity can grow in soils enriched with heavy metals such as Zn, Cu, Mn, and they can be absorbed and transferred to the senescent parts, as occurs in *Cupressus sempervirens* plants^40,41^.

A study evaluated the response of different plants, including ornamental, such as *Camelina sativa*, *Helianthus annuus*, *Festuca rubra*, *Amaranthus cruentus*, *Brassica napus*, *Melilotus albus*, *Beta vulgaris*, in soils with the addition of biosolids, containing high concentrations of metals such as Zn, and found that the plants were useful in soils strongly degraded by salts, and they even showed physical improvements from cultivation, indicating the possibility of plant use in environments with high salt concentrations^42^.

For copper accumulation, the study states that an accumulation of 5000 to 20,000 μg kg^−1^ is considered adequate for normal plant growth and, in cases of toxicity, changes manifest in the roots, which tend to lose vigor, acquire’ dark color, show thickening and paralyze growth^37^. Another research analyzed the accumulating potential of Cu in ‘Dark Fiji’ chrysanthemum plants, by adding 250, 500, 750 and 1000 mg kg^−1^ in the soil and observed a high copper accumulation in plant roots and low plant and flower development in all the analyzed doses, indicating that the chrysanthemum plant is tolerant to excess copper in soil and tissues, assuming potential phytoremediation characteristics^43^. According to the authors, considering copper accumulation in the roots, and being a heavy metal when at high concentrations, there is a mechanism of the root system that regulates the absorption of heavy metals, and leads to copper accumulation in the roots, thus having a low translocation to the shoot, contributing to the tolerance of the species to this metallic element.

As for boron accumulation, studies pointed out that the element acts in a plant defense mechanism to relieve aluminum toxicity, as it improves root growth and physiological characteristics of plants under aluminum toxicity, by regulating multiple physiological processes, activation of defense systems and reduction in oxidative damage. For the authors, the supply of B can reduce Al immobilization and restrict the entry of the element into the symplast, thus relieving its toxicity in environments with high aluminum concentrations^44^.

Regarding manganese accumulation, the effects of pine bark and biosolid residues were evaluated on nutrient accumulation in *Rosmarinus officinalis*, and it was observed that plants grown in pine bark showed lower growth and those submitted to biosolid residues accumulated a high concentration of Mn in the leaves, at contents of 106.6 mg kg^−1^, where it was possible to observe a slight leaf chlorosis, attributed by the high concentration of the element^24^.

It is possible to infer that the addition of 20% and 40% biosolids in the commercial substrate yielded the best nutritional contributions to the plants, highlighting the supply of P, K, Ca, Mg, S, B, Mo and Zn, which influenced the best photosynthetic rates and resulted in greater increases in dry biomass and number of flower buds produced. Among the analyzed organs, the leaves showed greater accumulation for the majority of the analyzed elements, P, K, Ca, Mg, S, Mn, Mo and B. Concentrations above 60% biosolid were not satisfactory, since they delayed the establishment and initial plant growth, with low dry biomass increments due to the less favorable conditions of these substrates, such as density, porosity, drainage and aeration, yielding, therefore, plants with lower quality standards.

## Material and methods

The biosolid was collected at the Sewage Treatment Plant (Coságua), set in Paraguaçu—MG. The biosolid was transported to the drying yard and thus dried with natural insolation, stirred daily for 4 days. After drying, a 400 g was collected for chemical determination according to Raij et al.^45^. The chemical composition of the biosolid and the commercial substrate are presented in Table 1 expressed in g kg^−1^ of macronutrients and mg kg^−1^ of micronutrients Table 1.

**Table 1:** Biosolid chemical composition and commercial substrate used in the experiment.

N	P	K	Ca	Mg	S	Fe	Mn	Zn	Cu	B	CTC
g kg^−1^						mg kg^−1^					mmolc dm^−3^
Biosolid											
30.3	4.7	2.9	21.4	2.3	5.6	14,462	120	395	107	47	759.7
Commercial substrate											
6.1	1.1	2.2	11.2	7.9	1.2	9433	122	21	12	29	571.7

The experimental design was in randomized blocks, with 6 treatments and 5 replicates; the replicate was the mean of the results of each chrysanthemum cutting in the pot. A total of 240 vessels (40 vessels/treatment) was used, and each vessel contained 3 chrysanthemum cuttings (720 in total). The treatments consisted of a homogeneous mixture based on the volume of commercial substrate + biosolid as a substrate for cultivation at the following concentrations: 100% pine bark; 20% biosolid + 80% pine bark; 40% biosolid + 60% pine bark; 60% biosolid + 40% pine bark; 80% biosolid + 20% pine bark and 100% biosolid. Data were submitted to analysis of variance and the means were compared by the Scott Knott test at 5% significance, using the Sisvar software^46^.

The experiment was set under greenhouse conditions, located at latitude 21° 27′ 11.54′′ S, longitude 45° 56′ 41.51′′ W and 848 m altitude, average temperature of 23 °C. Non-rooted chrysanthemum cuttings (‘White Diamond’) were purchased from Empresa Terra Viva LTDA and grown in black plastic pots with 8 holes and without dishes, with a volume of 1.3 L and 12 cm in height, containing growing substrate. The commercial substrate was purchased at Terra do Paraíso LTDA, and it consisted of 75% pine bark and 25% vermiculite, pH 6.5 and electrical conductivity of 0.50 mS cm-1.

Cutting planting was carried out in March 2017, directly on the growing substrate. Three cuttings per vessel, already treated with 0.1% indolebutyric acid (AIB) growth regulator, were arranged. In this period, to ensure that the substrate was maintained with good moisture in order to preserve the turgor of the newly planted cuttings and ensure their rooting, 150 mL water were applied per pot 2 to 3 times a day with the aid of a beaker, where they also received water directly in their structures through a manual sprayer, to maintain their hydration. Subsequently, during the experiment, the plants continued to receive water at amounts of 150 mL to 200 mL per day in each vessel, according to the development phases, with the aid of a beaker, always observing substrate moisture. Two weeks after planting, the pinch (breaking the apical dominance and stimulating lateral buds) was carried out, removing about 2 cm from the end, leaving 3 leaf pairs per plant.

During the 21-day period, from the date of planting, the cuttings were submitted to long days (DL) with 16 h day^−1^ of light, with the use of artificial lighting, using incandescent 60-W bulbs, installed at a height of 1.20 m from the pots and spaced 2 m apart from each other in the cyclic light system (alternating 15 min of lighting with 15 min of dark in the period from 9.00 p.m. to 4.00 a.m.). After 21 days of exposure to DL conditions on the third week of planting, the seedlings were submitted to short day conditions (DC), with 16 h day^−1^ of dark, from 4.00 p.m. to 8.00 a.m., performed with the use of black Oxford cloth with a double layer, arranged at a height of 1.00 m from the pots, thus covering the whole area of the experiment comprised by the pots. The application of short days through the cloth was interrupted from the observation of the appearance of flower buds. Subsequently, the lateral buds were removed as they became visible, keeping only the apical flower bud on each stem. The cultivation procedures were based on the commercial production of pot chrysanthemum. The cultivation period was 90 days and eight evaluations were carried out (one evaluation every week), from the 5th week until the end of the cultivation period (12th week). The results for the nutritional verification and accumulation of elements were taken only on the last week of cultivation.

In order to evaluate the rates of absorption, transport, accumulation and redistribution of elements in the different parts of plants, 15 plants were collected per treatment for 8 weeks, in a total of 90 plants per week of evaluation. The plants were separated according to each organ (root, stem, leaf and inflorescence), placed separately in properly identified kraft paper bags and then taken to an oven at 65 °C until constant weight. These procedures were performed weekly and with all 90 plants collected, generating a total of 2475 samples. The different organs of the same concentration were then mixed and homogenized, comprising a single sample, generating a total of 168 plant tissue samples. These samples were sent to the soil and plant tissue analysis laboratory of Cooperativa Regional de Cafeicultores em Guaxupé—Cooxupé, for determination and quantification of macro- and micronutrient concentrations and heavy metals in different organs. The N content in plant tissue was determined by the sulfur digestion method (Kjeldahl), phosphorus by spectrophotometry, calcium, magnesium and potassium and micronutrients by atomic absorption spectrophotometry.

For the analysis of net photosynthetic rate and transpiration, a portable CO2 and infrared gas analyzer (IRGA Li-6400, LI-COR Biosciences Inc., Nebraska, USA) was used. Five pots were evaluated in each treatment and one plant per pot, and the 4th leaf was duly marked from the apex. Readings were taken in the middle region of the leaf and in the morning, between 09.30 and 11.00 a.m.

Thirty plants were measured and the net photosynthetic rate per unit leaf area (A) and transpiration (E) were determined. The photosynthetically active radiation (RFA) and the concentration of atmospheric CO2 (Ca) were fixed to 1000 μmol photons m^−2^ s^−1^ and 400 μmol mol^−1^, respectively.

### Plant specimen collection declaration

We declare that the “White Diamond” species used in this biotest is a cultivated and commercial species, and is not a biotest with a wild plant. Therefore, it complies with the relevant institutional, national and international guidelines and legislation f or collecting plant material, in particular Law No. 9456, of April 25, 1997, which deals with the Cultivar Protection Law.

## Conclusions

It can be inferred that biosolids are a good source of nutrients in chrysanthemum cultivation, and in this study, concentrations of 20% to 40% added to the commercial substrate showed the best results, and it should be verified which elements and concentrations are present in the composition and new studies be carried out on other plant species.

## Data Availability

The data is stored and made available in DOCX, PDF and JPEG formats, among others, according to each stage of execution of the work. Access will be preserved through the Theses/Dissertations repository of the Federal University of Alfenas, which is open to consultation by the entire scientific community. Any references in literature must respect the credit of the author of the published data or result. The researchers involved are responsible for the data, backup of the activities, for the production of metadata as well as their quality. Data are stored in a free and easily accessible repository to make them available quickly and efficiently to interested parties. Direct accessible link for the repository: https://www.unifal-mg.edu.br/ppgca/wp-content/uploads/sites/188/2021/03/Dissertacao_Frederico-Luiz-Pereira-1.pdf. https://www.unifal-mg.edu.br/ppgca/teses-e-dissertacoes/
